# Role of Artificial Intelligence and Machine Learning in Conservative Dentistry and Endodontics: A Review

**DOI:** 10.7759/cureus.88515

**Published:** 2025-07-22

**Authors:** Rajinder K Bansal, Ashtha Arya, Birmohan Singh, Mamta Singla, Seema Gupta

**Affiliations:** 1 Department of Conservative Dentistry and Endodontics, Shree Guru Govind Singh Tricentanary Dental College, Hospital and Research Institute, Gurugram, IND; 2 Department of Computer Science and Engineering, Sant Longowal Institute of Engineering and Technology, Longowal, IND; 3 Department of Orthodontics, Kothiwal Dental College and Research Centre, Moradabad, IND

**Keywords:** applications, artificial intelligence, conservative dentistry, deep learning, machine learning

## Abstract

Artificial intelligence (AI) and machine learning (ML) have emerged as transformative tools in conservative dentistry and endodontics, revolutionizing diagnostic accuracy, treatment planning, and procedural efficiency. This narrative review explores the applications, methodologies, advantages, and challenges of AI in these fields. AI-driven systems, such as convolutional neural networks (CNNs), excel in analyzing dental imaging, including radiographs and cone-beam computed tomography, to detect caries, periapical lesions, and root canal morphologies with high precision. These technologies streamline tasks such as tooth shade determination and working length measurement, reducing human error and enhancing clinical outcomes. Predictive models utilize patient data to assess the risks of caries progression and endodontic complications, thereby enabling the development of personalized treatment plans. Natural language processing aids in extracting insights from clinical records, while generative adversarial networks enhance dataset quality by creating synthetic images. Despite these advancements, challenges persist, including limited availability of diverse, annotated datasets, which affects model generalizability across populations. The opaque nature of some AI algorithms raises concerns about interpretability, potentially undermining clinician trust. High computational requirements and implementation costs limit accessibility, particularly in resource-constrained settings. Ethical issues, such as patient data privacy and the risk of over-reliance on AI, further complicate adoption. Addressing these barriers requires standardized dental imaging databases, transparent algorithms, and collaboration between dental professionals and data scientists. Future research should focus on improving model explainability, expanding dataset diversity, and integrating AI seamlessly into clinical workflows. By overcoming these challenges, AI and ML hold the potential to become indispensable in conservative dentistry and endodontics, offering precise, efficient, and patient-centered solutions that enhance diagnostic reliability and treatment success, ultimately advancing the quality of dental care. This narrative review aimed to explore the theoretical foundations, historical evolution, and practical applications of AI and ML in conservative dentistry and endodontics, with a focus on their types, methodologies, advantages, and limitations.

## Introduction and background

Conservative dentistry and endodontics focus on preserving natural tooth structure through the prevention, diagnosis, and treatment of dental caries, pulp diseases, and periapical pathologies [1]. The integration of advanced computational technologies, such as artificial intelligence (AI) and machine learning (ML), has revolutionized these fields by enhancing diagnostic accuracy, treatment planning, and procedural outcomes [2]. AI and ML systems analyze complex datasets, including radiographic images, clinical records, and patient histories, to identify patterns and predict outcomes with precision. For instance, these technologies have been applied to detect caries, assess root canal morphology, and determine working lengths, thereby reducing human error and improving clinical decision-making [3,4].

Studies have demonstrated that AI-based tools can achieve diagnostic sensitivity and specificity comparable to or exceeding that of experienced clinicians, as noted in a systematic review by Khanagar et al. [5]. Furthermore, ML algorithms, particularly deep learning models, have been employed to automate tooth segmentation and shade determination, streamlining restorative procedures [6]. The potential of AI and ML extends to personalized treatment planning, where predictive models assess the risk of caries progression or endodontic complications based on patient-specific factors [7].

Despite these advancements, challenges such as data quality, ethical concerns, and the need for robust validation remain significant barriers to widespread adoption [7]. The literature highlights the importance of interdisciplinary collaboration between dental professionals and data scientists to develop reliable AI systems tailored to clinical needs [8,9]. This narrative review aimed to explore the theoretical foundations, historical evolution, and practical applications of AI and ML in conservative dentistry and endodontics, with a focus on their types, methodologies, advantages, and limitations.

## Review

Literature search strategy

A systematic literature search was conducted to identify relevant studies on the applications of AI and ML in Conservative Dentistry and Endodontics. The search was performed across multiple electronic databases, including PubMed, Scopus, Web of Science, and Google Scholar, covering publications from January 2000 to October 2024. This time frame was selected for the literature search to capture the emergence and evolution of AI and ML in healthcare, particularly in dentistry, as computational advancements enabled early applications like support vector machines. This timeframe ensures inclusion of foundational studies and recent developments, covering the rapid growth of AI in conservative dentistry and endodontics while aligning with the review’s aim to explore historical and current trends.

The following keywords and their combinations were used: "artificial intelligence," "machine learning," "deep learning," "convolutional neural networks," "recurrent neural networks," "generative adversarial networks," "natural language processing," "computer vision," "conservative dentistry," "endodontics," "caries detection," "periapical lesions," "root canal morphology," "tooth shade determination," and "working length determination." Boolean operators (AND, OR) were employed to refine the search.

Inclusion and exclusion criteria

Studies were included if they met the following criteria: peer-reviewed articles, conference papers, or systematic reviews published in English; focused on AI or ML applications in conservative dentistry or endodontics; addressed diagnostic, predictive, or operational applications, including caries detection, periapical lesion identification, root canal morphology, tooth restoration, or working length determination; and provided empirical data or theoretical insights on AI/ML methodologies. Exclusion criteria included: non-peer-reviewed sources, such as editorials or opinion pieces; studies not specific to dentistry or endodontics; studies lacking a clear focus on AI or ML; and non-English publications.

Study selection process

The search yielded an initial pool of articles, which were screened in three stages. First, titles and abstracts were reviewed to assess relevance based on inclusion criteria. Second, full-text articles were retrieved for potentially relevant studies and evaluated for methodological rigor and relevance to AI/ML in dentistry. Third, reference lists of selected articles were manually searched to identify additional relevant studies (snowballing technique). Two independent reviewers conducted the screening process, and discrepancies were resolved through discussion or consultation with a third reviewer.

Data extraction

Data from selected studies were extracted using a standardized template, capturing the following information: study objectives, AI/ML techniques used, applications in conservative dentistry or endodontics, dataset characteristics, advantages reported, limitations identified, and future recommendations. Data were organized into thematic categories, including theoretical foundations, historical context, types of AI/ML, and clinical applications.

Data synthesis and analysis

A narrative synthesis approach was employed to summarize findings, given the heterogeneity of study designs and AI/ML methodologies. The review was structured into sections addressing: theoretical foundations of AI and ML, historical evolution of AI in healthcare and dentistry, types of AI (based on capability, function, and healthcare applications), ML and deep learning methodologies, specific applications in conservative dentistry and endodontics, advantages, and limitations. Key findings were synthesized to highlight trends, such as the dominance of convoluted neural networks (CNNs) in caries detection, and gaps, such as the need for standardized dental imaging datasets. The review examined ethical issues in AI/ML applications, such as data privacy, algorithmic bias, and clinical interpretability, as reported in the literature. These were integrated into the discussion of limitations and future directions, emphasizing the need for transparent algorithms and patient data security.

Artificial intelligence (AI)

AI refers to the development of computational systems capable of performing tasks that typically require human cognitive abilities, such as reasoning, problem-solving, and decision-making [10]. In dentistry, AI systems process large datasets, including images and clinical records, to identify patterns and make predictions. These systems rely on algorithms that mimic human intelligence, enabling applications like automated diagnosis and treatment planning [2]. AI encompasses various subfields, including ML, natural language processing, and computer vision, each contributing to its functionality. In Conservative Dentistry and Endodontics, AI enhances diagnostic precision and procedural efficiency [7,9,11].

History of AI

**Figure 1:**
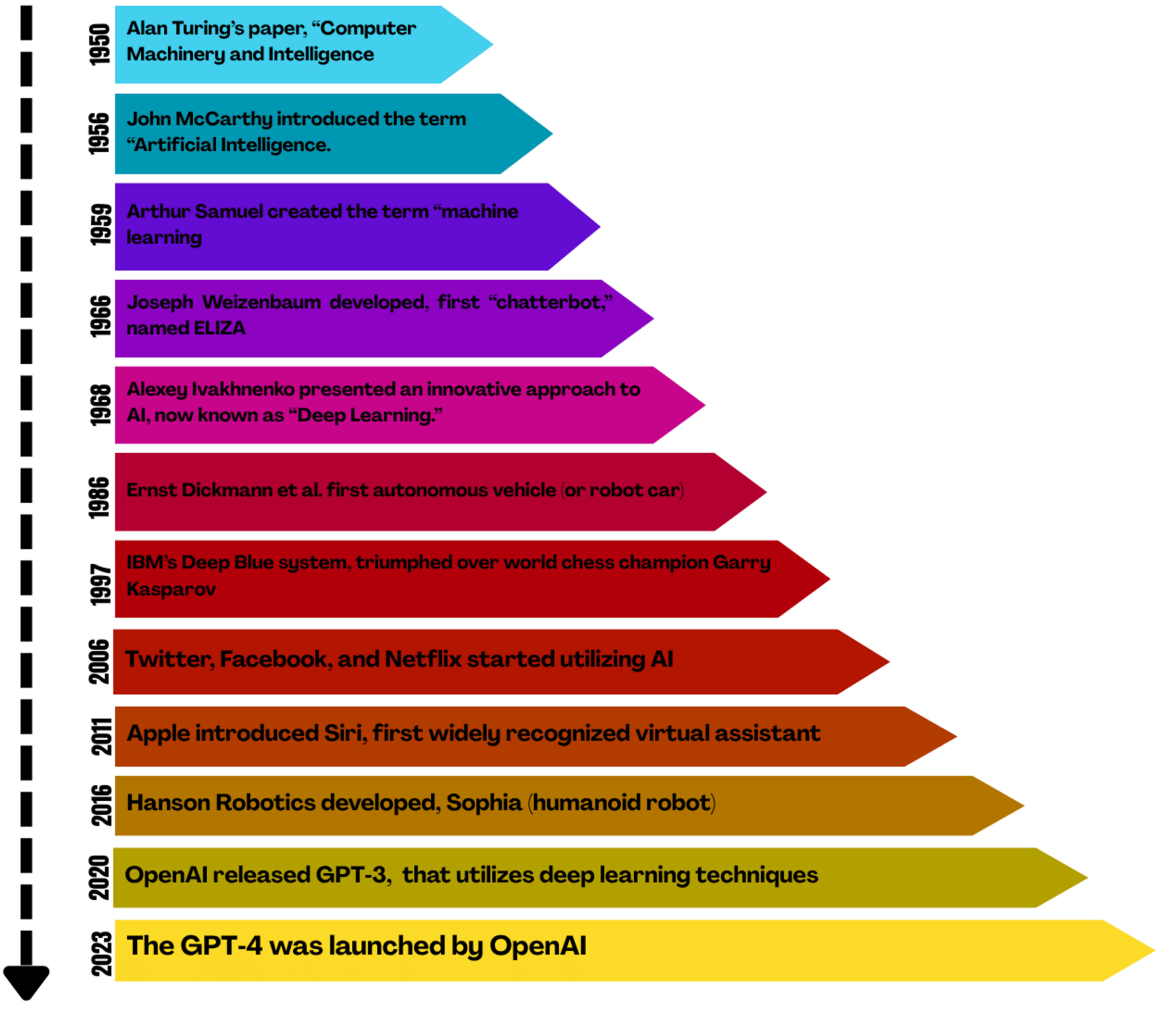
History of artificial intelligence. This image is created by author.

The application of AI in health sciences began in the 1960s (Figure 1) with the development of expert systems like INTERNIST-I (1970s), which provided multiple and complete diagnoses in internal medicine [12]. These early systems relied on rule-based algorithms but were limited by their inability to handle uncertainty. The 2000s marked a turning point with the advent of ML, enabling the analysis of large datasets, as seen in the use of support vector machines for cancer classification [13]. In dentistry, AI gained traction in the 2010s, with previous studies demonstrating AI’s potential in detecting periapical lesions [14,15]. Today, AI applications in health sciences, including dentistry, leverage deep learning and big data to enhance diagnostics, treatment planning, and patient outcomes.

Types of AI

AI can be classified based on capability into three categories: narrow, general, and superintelligence. Narrow AI, the most prevalent type, is designed for specific tasks, such as caries detection in dental radiographs, where it outperforms human experts in sensitivity [16]. General AI, which aims to replicate human cognitive abilities across various tasks, remains theoretical and is not yet realized. Superintelligence, a hypothetical future concept, would surpass human intelligence, raise ethical concerns, but offer transformative potential in healthcare [9].

Functionally, AI is categorized into reactive, limited memory, theory of mind, and self-aware systems. Reactive AI, like early diagnostic tools, responds to inputs without memory, limiting its adaptability. Limited memory AI, such as ML models in endodontics, uses historical data to improve predictions, as seen in working length determination [17]. Theory of mind AI, which understands human emotions, and self-aware AI, which possesses consciousness, are not yet developed but could eventually enhance patient interactions in dental practice.

Application of AI in healthcare

**Figure 2:**
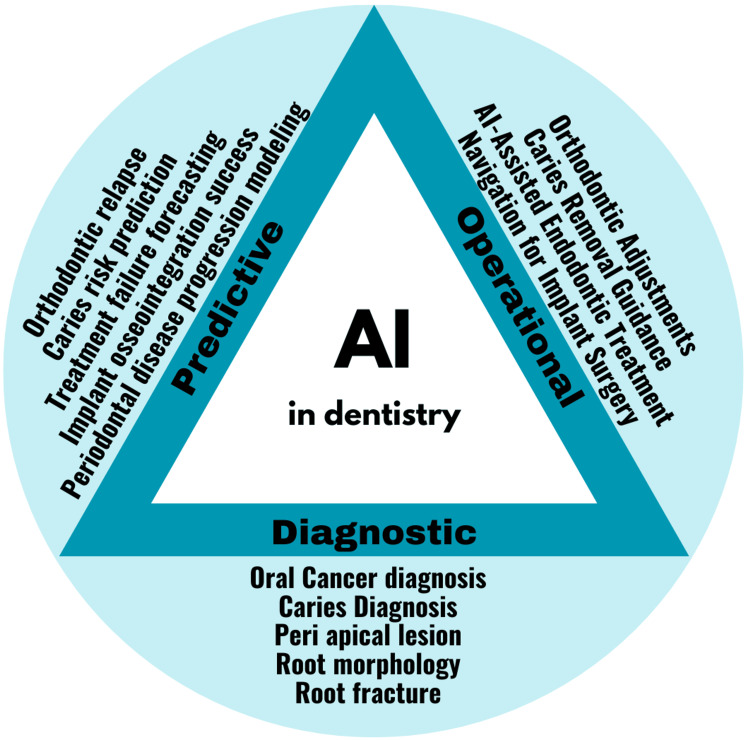
Uses of artificial intelligence in dentistry. This image is created by author.

In healthcare, AI applications include diagnostic, predictive, and operational systems. Diagnostic AI, widely used in dentistry, analyzes radiographic images to detect pathologies like periapical lesions with high accuracy [15]. Currently, three methodologies utilizing artificial intelligence have been introduced for the identification of dental caries. These methodologies encompass image-based caries detection employing AI, AI-facilitated assessment of caries risk, and the amalgamation of artificial intelligence with Computer-Aided Diagnosis (CAD) systems [4]. Predictive AI models assess risk factors, such as caries progression, with 97.1% precision, by analyzing patient data, as demonstrated by Hung et al. [18]. Operational AI streamlines administrative tasks, such as appointment scheduling, indirectly benefiting clinical workflows. In conservative dentistry and endodontics, these applications improve diagnostic precision, optimize treatment planning, and enhance patient care, though their effectiveness depends on data quality and algorithmic robustness (Figure 2).

Machine learning

ML is a subfield of AI that enables systems to learn from data and improve performance without explicit programming. ML algorithms identify patterns in datasets, making them suitable for tasks like predicting dental caries risk or classifying root canal morphologies [17,18]. In conservative dentistry, ML models analyze clinical and imaging data to support decision-making, as evidenced by studies such as those by Herbst et al., which used CNNs to detect periapical lesions with 89-90% accuracy [19]. ML systems rely on training datasets to develop models, which are then validated on unseen data to ensure generalizability. The process involves feature extraction, model training, and performance evaluation using metrics like sensitivity and specificity. In endodontics, ML has been applied to predict the prognosis of endodontic microsurgery [20] and for predicting failure of root canal treatment [21]. Unlike traditional statistical methods, ML can handle non-linear relationships and high-dimensional data, making it ideal for complex dental applications. However, its effectiveness depends on the quality and quantity of training data, as well as the selection of appropriate algorithms, highlighting the need for standardized datasets in dental research.

There are four types of ML: supervised, unsupervised, semi-supervised, and representation learning. Supervised learning involves training models on labeled datasets, where input-output pairs are provided. In dentistry, semi-supervised learning is used to classify dental caries in radiographs, with algorithms that have provided performance improvement of approximately 6% and 3% in terms of average pixel accuracy [22]. The model learns to map inputs (e.g., pixel intensities) to outputs (e.g., caries presence), making it suitable for diagnostic tasks.

Unsupervised learning identifies patterns in unlabeled data, often through clustering or dimensionality reduction. In endodontics, unsupervised methods such as k-means clustering have been used to group root canal morphologies based on cone beam computed tomography (CBCT) images, revealing anatomical variations [23]. This approach is valuable for exploratory analysis but may lack interpretability without ground truth labels.

Semi-supervised learning combines labeled and unlabeled data, addressing the challenge of limited labeled datasets in dentistry. For instance, semi-supervised algorithms have been applied to detect periapical lesions, leveraging small labeled datasets and larger unlabeled CBCT scans to improve model performance [24]. This method balances accuracy and scalability, making it practical for clinical applications.

Representation learning automatically extracts features from raw data, reducing the need for manual feature engineering. In conservative dentistry, representation learning has been used to identify tooth preparation margins by transforming radiographic images into meaningful features, as demonstrated by Hung et al. [18]. This approach enhances model robustness, particularly for complex tasks such as tooth shade determination, where traditional feature extraction may fail to capture subtle variations. Each ML type offers unique benefits in dentistry, from supervised learning’s precision in diagnostics to unsupervised learning’s ability to uncover hidden patterns, though their effectiveness depends on data availability and quality.

Deep learning

Deep learning (DL), a subset of ML, utilizes neural networks with multiple layers to analyze complex data. In conservative dentistry and endodontics, DL excels in image-based tasks, such as detecting caries and periapical lesions, due to its ability to learn hierarchical features directly from raw data [25-30]. DL models, such as convolutional neural networks (CNNs), process large datasets such as CBCT images to identify anatomical structures, including root canal morphology [28]. Unlike traditional ML, DL does not require manual feature engineering, making it efficient for high-dimensional data. However, DL demands substantial computational resources and large, annotated datasets, which can be a limitation in dentistry due to the scarcity of standardized dental imaging databases. Additionally, the "black box" nature of DL models raises concerns about interpretability in clinical decision-making, necessitating further research into explainable algorithms for dental applications.

An artificial neural network (ANN) is composed of numerous diminutive communication units referred to as neurons, which are systematically organized into distinct layers. This widely recognized category of algorithms encompasses an input layer, an output layer, and multiple intermediate hidden layers. Based on the quantity of hidden layers present, a neural network may be characterized as either shallow (containing one or a limited number of hidden layers) or deep (incorporating numerous hidden layers, commonly referred to as a deep neural network) [24].

Deep neural networks (DNNs) contain multiple hidden layers, enabling them to learn intricate patterns in high-dimensional data. In endodontics, DNNs have been applied to detect periapical pathosis in CBCT images, achieving 92.8% accuracy [25]. DNNs excel in tasks requiring hierarchical feature extraction, such as identifying root canal morphology, but require large datasets and computational power. Their complexity also poses challenges in interpreting decision-making processes, which is critical for clinical acceptance in conservative dentistry and endodontics.

Convolutional Neural Networks (CNNs) are a specialized type of deep neural network designed for processing structured grid-like data, such as images, making them highly effective in dental imaging applications. CNNs consist of convolutional layers that apply filters to extract features (e.g., edges, textures), followed by pooling layers that reduce spatial dimensions while preserving essential information [26]. In conservative dentistry, CNNs have been widely used for caries detection with 82% accuracy [27], to classify C-shaped canal anatomy in mandibular second molars from CBCT volumes [28], for detecting vertical root fracture on panoramic radiography with 93% precision [29], and for radiographic detection of periapical lesions with 95% accuracy [30].

The strength of CNNs lies in their ability to learn spatial hierarchies of features directly from raw images, eliminating the need for manual feature engineering. However, their performance depends on large, annotated datasets, which are often limited in dentistry due to privacy concerns and high imaging costs. Additionally, CNNs require significant computational resources, and their lack of interpretability can hinder clinical adoption. Despite these challenges, CNNs remain a powerful tool for enhancing diagnostic precision and procedural outcomes in conservative dentistry and endodontics, with ongoing research focusing on improving model transparency and generalizability [26].

Recurrent Neural Networks (RNNs) are designed to process sequential data by maintaining a "memory" of previous inputs through feedback loops, making them suitable for tasks involving temporal or sequential patterns [31]. In conservative dentistry and endodontics, RNNs have been applied to analyze time-series data, such as patient treatment histories or sequential radiographic changes. For example, RNNs can predict caries progression by evaluating longitudinal clinical records, as shown in a study by Hung et al., which achieved 87% accuracy in forecasting caries risk over a 12-month period [18]. However, traditional RNNs suffer from issues like vanishing gradients, which limit their ability to capture long-term dependencies. Variants like Long Short-Term Memory (LSTM) units address this by selectively retaining information over extended sequences, improving performance in dental applications. Despite their potential, RNNs are underutilized in dentistry due to the scarcity of sequential dental datasets and the computational complexity of training such models [31].

Generative Adversarial Networks (GANs) consist of two models, a generator and a discriminator, that are trained simultaneously in a competitive setting. The generator creates synthetic data, while the discriminator evaluates its authenticity, leading to the production of realistic outputs [31]. Kim et al. employed masking techniques to eliminate the interdental space, subsequently utilizing a GAN to reconstruct the boundary outlines [32]. The proposed methodology resulted in an enhancement of precision to 0.004 mm compared to isolated scanning, which excluded interdental regions. However, due to the occlusion of adjacent normal anatomical structures, the dimensions of the mask exhibited a negative correlation with the accuracy of the reconstruction. Kokomoto et al. illustrated the generation of full-color intraoral images through the progressive growth of Generative Adversarial Networks (PGGAN) and evaluated both the quantity and visual quality of the resultant intraoral images as assessed by pediatric dental practitioners [33]. However, GANs are computationally intensive and prone to mode collapse, where the generator produces limited varieties of outputs. Their application in dentistry is promising but requires further validation to ensure the clinical reliability of synthetic data in diagnostic and treatment contexts.

Applications of AI in conservative dentistry and endodontics

Detection of Caries

Al-Khalifa et al. conducted a review to assess the role of AI in the detection of caries and concluded that AI systems, particularly CNNs, have been extensively applied to detect dental caries with high accuracy [4]. Nevertheless, the identification of dental caries in teeth exhibiting intricate morphological characteristics, such as molars, continues to pose significant challenges for these analytical models. For example, a research investigation focusing on the identification of caries utilizing a CNN model illustrated a superior accuracy of 91% for premolars and 89% for molars in recognizing caries in premolars relative to molars, which possess a complex morphology [34]. Both ML and Deep Learning (DL) are regarded as efficacious methodologies for the diagnosis and prognostication of dental caries risk. The efficacy of these applications is significantly contingent upon the provision of appropriate and comprehensively annotated datasets [22]. By automating caries detection, AI reduces diagnostic variability and enhances treatment planning in conservative dentistry; however, its performance depends on the quality of training data and image resolution.

Identification of Tooth Preparation Margins

AI facilitates the identification of tooth preparation margins in restorative dentistry by analyzing intraoral scans and radiographic images. ML models, such as representation learning, extract features like margin contours, achieving 89% accuracy in margin detection [35]. This application ensures precise preparation for crowns and fillings, thereby improving restoration fit and longevity. AI’s ability to standardize margin identification reduces operator variability, enhancing procedural outcomes in conservative dentistry, though challenges remain in handling complex tooth geometries.

Detection of the Type of Restoration

AI has been employed to detect and evaluate tooth restorations, such as fillings and crowns, using computer vision and deep learning techniques. AI analyzes radiographs to identify the presence, type, and integrity of restorations, using automatic segmentation technique achieving high accuracy [36]. AI also assesses restoration margins for defects, identifying secondary caries or marginal gaps that may require intervention. This application enhances the longevity of restorations by enabling early detection of failures, though its effectiveness is limited by the variability in restoration materials and imaging artifacts, necessitating further research into robust algorithms.

Tooth Shade Determination

AI-based systems automate tooth shade determination by analyzing intraoral images to match shades with standardized charts, such as the Vita shade guide. Shetty et al. executed a systematic review aimed at evaluating the precision of artificial intelligence in the domain of dental shade selection [37]. The review encompassed 53 scholarly articles and identified that the artificial intelligence algorithms employed for shade-matching comprised fuzzy logic, a genetic algorithm integrated with back-propagation neural networks, back-propagation neural networks, CNN, ANN, support vector machine algorithms, K-nearest neighbor in conjunction with decision tree methodologies, and random forest techniques. The decision tree regression model exhibited the highest accuracy in predicting dental shades for leucite-based dental ceramics, achieving an impressive accuracy rate of 99.7%, followed closely by fuzzy logic with an accuracy of 99.62%, and the support vector machine employing cross-validation, which attained an accuracy rate of 97%.

Detection of Root Canal Morphology

AI, particularly CNNs, has been used to detect root canal morphology in CBCT images, identifying canal configurations. Despite the CNNs attaining a commendable accuracy rate of 86.9%, the incorporation of this technology into clinical practice is accompanied by various challenges [38]. Primarily, the process of image segmentation necessitates manual intervention, which is labor-intensive [38]. In addition, it is imperative that the images are of sufficient size while concurrently concentrating on a particular area and encompassing an adequate context to incorporate all pertinent information regarding the subject of examination [39]. These models segment canal structures, aiding in treatment planning by revealing anatomical variations, such as accessory canals [28]. This application reduces procedural errors in endodontics, improving outcomes, though it requires high-quality imaging and large datasets for training, which may not always be available.

Detection of Periapical Lesions

AI has significantly advanced the detection of periapical lesions, a critical aspect of endodontic diagnosis. CNNs and deep learning models analyze panoramic and CBCT images to identify periapical radiolucencies, achieving sensitivity rates of 92.5% [14]. A study by Orhan et al. reported that AI models could differentiate between periapical lesions and other radiolucencies, such as cysts, with 90% specificity [25]. These systems assist clinicians in early diagnosis, enabling timely intervention to prevent complications like bone loss. AI also quantifies lesion size and monitors progression over time, supporting treatment evaluation. For instance, Calazans et al. demonstrated 68-70% accuracy of CNNs in classifying periapical lesions [40]. Ketenci et al. demonstrated 90% accuracy of AI in detecting and segmenting apical lesions on panoramic radiographs [41]. However, challenges include distinguishing between healing lesions and persistent pathology, as well as the need for standardized imaging protocols. Despite these limitations, AI’s high diagnostic accuracy makes it a valuable tool in endodontics, with potential to improve patient outcomes through precise and consistent lesion detection.

Determination of Vertical Root Fracture

A study conducted by Fukuda et al. indicated that a CNNs might serve as a valuable instrument for the detection of vertical root fractures in panoramic radiographs [29]. These models identify fracture lines that are often missed in conventional radiographs, aiding in early diagnosis and treatment planning. AI’s ability to detect vertical root fractures improves prognostic accuracy in endodontics, though its performance is limited by image resolution and the need for large, annotated datasets, which are scarce for this specific application.

Working Length Determination and Detection of Apical Foramen

AI has been utilized to determine working length and detect the minor apical foramen in endodontics using radiographic images. Saghiri et al. indicated that the application of ANNs to offer an auxiliary assessment concerning the positioning of the radiographic apical foramen can enhance the precision of working length measurements [42]. A related investigation by Saghiri et al., published in the same year, scrutinized the accuracy of working length assessment through an ANN utilizing a human cadaver model to replicate a clinical environment [43]. The investigators discovered no statistically significant disparity in root length measurements between the ANN’s findings and the actual measurements post-extraction. Moreover, they documented that the ANN, exhibiting an accuracy rate of 96%, notably surpassed an endodontist (76%) in recognizing minor anatomical constrictions via IOPA [42]. Consequently, ANNs seemingly yield more accurate determinations of working length compared to endodontist evaluations when employing IOPA. This application enhances the success of root canal treatments, though it requires high-quality imaging and standardized protocols to achieve reliable results.

Challenges in the use of AI in conservative dentistry and endodontics

The integration of AI faces significant challenges that hinder its widespread adoption. A primary issue is the scarcity of large, high-quality, annotated datasets, as dental imaging data are often limited due to privacy concerns and high acquisition costs, leading to models that may fail to generalize across diverse populations [5]. The "black box" nature of deep learning models, such as convolutional neural networks, reduces interpretability, making it difficult for clinicians to trust AI-driven diagnoses [9]. High computational requirements and implementation costs further restrict accessibility, particularly in resource-limited settings, while ethical concerns, including data security and potential over-reliance on AI, risk compromising clinical judgment [9]. Additionally, AI systems may struggle with rare or atypical cases, such as complex root canal morphologies, due to underrepresentation in training data, potentially leading to diagnostic errors. Addressing these challenges requires standardized datasets, transparent algorithms, and interdisciplinary collaboration to ensure AI complements clinical expertise effectively.

## Conclusions

AI and ML have revolutionized the field of conservative dentistry and endodontics by enhancing diagnostic precision, streamlining treatment planning, and improving procedural outcomes. These technologies excel in tasks like detecting dental caries, analyzing root canal morphology, and determining working lengths, often achieving high accuracy and consistency. AI-driven tools also support personalized treatment by predicting risks and optimizing restorative processes, ultimately improving patient care and clinical efficiency. However, challenges such as limited access to diverse dental datasets, high computational demands, and concerns over algorithm transparency pose barriers to widespread adoption. Ethical issues, including patient data privacy and the risk of over-relying on AI, further complicate integration into routine practice. Moving forward, addressing these hurdles through standardized data collection, improved algorithm interpretability, and collaboration between dental professionals and technologists will be crucial. By overcoming these obstacles, AI and ML have the potential to become integral to conservative dentistry and endodontics, offering innovative solutions that enhance accuracy, reduce errors, and deliver tailored, patient-centered care, thereby transforming the field for both practitioners and patients.
